# Effect of Ligand Substitution on the Formation of the Meltable Fe-ZIF

**DOI:** 10.3390/ma19101926

**Published:** 2026-05-08

**Authors:** Liuyang Zheng, Chaohui Guo, Zijuan Du, Juan Han, Ang Qiao, De Fang, Haizheng Tao

**Affiliations:** 1 State Key Laboratory of Silicate Materials for Architectures, Wuhan University of Technology, Wuhan 430070, Chinathz@whut.edu.cn (H.T.); 2 School of Materials Science and Engineering, Wuhan University of Technology, Wuhan 430070, China; juanh@whut.edu.cn

**Keywords:** meltable, metal–organic frameworks, synthesis, substitution

## Abstract

Meltable metal–organic frameworks (MOF) are essential for the formation of MOF glasses, which have emerged as a new family of functional materials offering promising potential for applications in gas separation, luminescence, energy storage, and beyond. Herein, the synthesis of iron-based zeolitic imidazolate framework (ZIF) crystals, specifically Fe_3_(Im)_6_(HIm)_2_, where Im is imidazolate, is reported. Upon the substitution of some Im linkers with a secondary ligand, 5,6-dimethylbenzimidazole (dmbIm), it was found that such substitution induces the formation of new phases: one phase exhibits meltability and subsequent glass formation, while another phase [Fe_3_(Im)_1.56_(dmbIm)_4.44_(HIm)_2_] is non-meltable. Through structural characterizations, the configuration of the tetrahedral [Fe-linkers] units was revealed to be crucial in determining the meltability of Fe-ZIF. The incorporation of a large secondary ligand hinders the occurrence of melting. This work provides an insight into how ligands affect the accessibility of the liquid state of MOFs, showing a practical strategy for designing meltable MOFs.

## 1. Introduction

Metal–organic frameworks (MOFs), a new class of functional materials, have garnered widespread attention owing to their exceptional structural and property tunability [1]. Precise modulation of metal nodes and organic linkers allows for the tailoring of their intrinsic physicochemical properties for applications in gas separation [2], storage [3], and catalysis [4]. Recently, the discovery of melting and vitrification in a subset of MOFs, i.e., zeolitic imidazolate frameworks (ZIFs), has opened new avenues for the preparation of new types of functional MOF materials [5,6]. MOF glasses offer superior processability and thermal stability [7], showing advantages in applications such as photonics [8] and gas separation [9].

Despite these advancements, the number of vitrifiable MOFs remains limited. The main difficulty is that for the vast majority of MOFs, the decomposition temperature (*T*_d_) precedes the theoretical melting point (*T*_m_), precluding access to a stable liquid phase [10]. In recent years, successful glass formation has been predominantly found in a narrow range of zeolitic imidazolate frameworks (e.g., ZIF-4, ZIF-62) [5]. To address this challenge, researchers have made efforts in the following directions. On the one hand, new synthesis methods such as melting under high pressure or mechanochemistry have been employed to depress *T*_m_ and expand the temperature range between *T*_m_ and *T*_d_ [11,12]. On the other hand, the metal node substitution or mixed-linkers strategies have also been used to regulate the thermodynamic behaviors of the framework [13]. For example, Thorne et al. successfully tuned the thermal properties of ZIF-62 by constructing a Zn/Co mixed-metal framework via mechanochemistry, demonstrating that the incorporation of up to 20% Co^2+^ within the framework resulted in a lowering of the *T*_m_ [13]. Ríos Gómez et al. synthesized multicomponent ZIF-UC-1 by introducing a third linker (5-methylbenzimidazole, mbIm) into the ZIF-62 system, revealing the correlation between increased configurational disorder and the resulting reduction in *T*_m_ and *T*_g_ shifts [14].

Concerning the effect of mixed-linker on the melting and glass forming of ZIFs, it is reported that incorporation of secondary linkers induces the distortion of [metal-ligands] tetrahedral units, diminishing the network cohesive interactions in the framework and hence leading to the decrease in *T*_m_ [15,16]. If the added secondary linker is larger than the initial one, it would also cause a steric hindrance effect that makes the crystalline lattice become rigid, thereby precluding melting [14]. When the added secondary linkers exceed the accommodation capacity of the crystal structure, it would trigger a structural transition to a new crystalline phase [15]. Taking the ZIF-4/ZIF-62 system as an example, Frentzel-Beyme et al. achieved a linear tuning of the *T*_m_ by adjusting the molar ratio of the Im ligand to the bulky benzimidazole (bIm) ligand. As the bIm doping ratio is gradually increased from 0.03 to 0.35, *T*_m_ decreases accordingly from approximately 440 °C to 370 °C. However, the complete substitution of Im with bIm results in the formation of non-meltable ZIF-7 (Zn(bIm)_2_) [15]. Huanni Xia et al. successfully obtained the TIF-4 mixed-linker framework by incorporating the bulky 5-methylbenzimidazole (5-mbIm) into the ZIF-4 framework. Compared to ZIF-4, the substitution with 5-mbIm resulted in a significant decrease in the melting point. However, as the molar fraction of 5-mbIm increased from 2.5% to 33%, the Tm gradually rose from 377 °C to 460 °C [17]. Therefore, the mixed-linkers strategy is feasible to tune the melting behavior of ZIF. However, how the linker substitution affects the demarcation between meltable and non-meltable ZIFs is not yet well understood.

**Figure 1:**
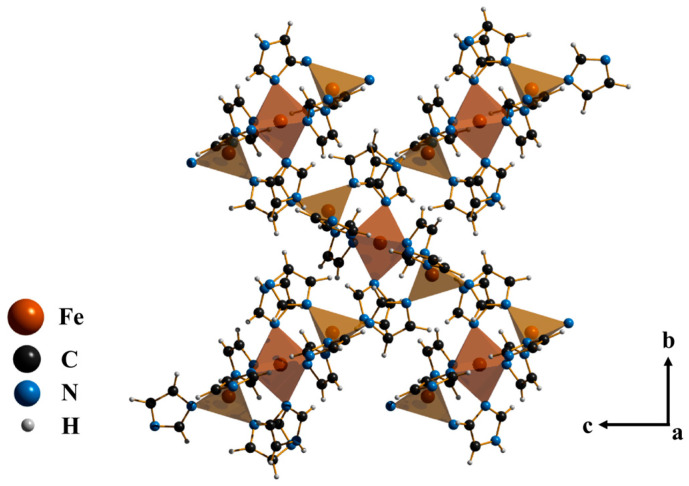
Schematic representation of the crystal structure of Fe-ZIF (Fe_3_(Im)_6_(HIm)_2_).

In this work, we synthesized a Fe-ZIF (Figure 1), i.e., Fe_3_(Im)_6_(HIm)_2_, where Im is imidazole and a series of Fe-ZIF samples with the substitution of Im by a large-size secondary linker of 5,6-dimethylbenzimidazole (dmbIm) via a solvent-free synthesis method. By using the Powder X-ray Diffraction (PXRD), Scanning Electron Microscopy (SEM), we investigated the crystalline phases in the products with the increase in dmbIm content and distinguished the meltable and non-meltable phases via Differential Scanning Calorimetry (DSC) measurement. Finally, the chemical components in different phases were detected through solution ^1^H Nuclear Magnetic Resonance (NMR) spectroscopy.

## 2. Materials and Methods

### 2.1. Materials

Ferrocene (Fc, 99%), imidazole (Im, 99%), 5,6-dimethylbenzimidazole (dmbIm, 99%), and *N*, *N*-dimethylformamide (DMF, 99.9%) were all purchased from Aladdin Reagent Co., Ltd. (Shanghai, China). All chemical reagents were of analytical grade and used without further purification.

### 2.2. Synthesis

**Figure 2:**
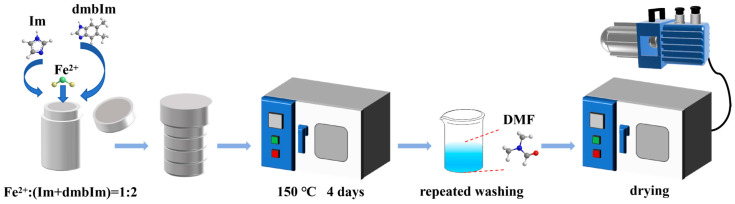
Procedure of crystal synthesis.

The experimental procedure of Fe-ZIF crystal synthesis is schematically illustrated in Figure 2. The molar ratio of Fc: Im:dmbIm was fixed at 0.01:(0.02-R):R, with R values of 0, 0.002, 0.003, and 0.004. The reagents were fully mixed and then loaded into a Teflon-lined autoclave. Subsequently, the hydrothermal reactor was sealed and heated in an oven at 150 °C for 96 h. After cooling to room temperature, the products were washed three times with DMF and dried under vacuum at 80 °C for 12 h. Moreover, the obtained samples were designated as FCIM, d_0.1_-FCIM, d_0.15_-FCIM, and d_0.2_-FCIM, corresponding to the ratio of added dmbIm concentration to the total ligand concentration (α = R/0.02) of 0, 0.1, 0.15, and 0.2 in synthesis, respectively.

### 2.3. Measurements

DSC measurements were performed using a NETZSCH STA 449F1 (NETZSCH, Selb, Germany) instrument under an argon atmosphere. Approximately 15 mg samples were placed in a platinum crucible situated on a sample holder of the DSC at room temperature. The samples were held for 10 min at an initial temperature of 40 °C, heated at 10 °C min^−1^ to the target temperature, and then cooled to 200 °C at 20 °C min^−1^. After natural cooling to room temperature, the second upscan was performed using the same procedure. Additionally, to prepare heat-treated specimens for further characterization, the FCIM series, Phase-1, and Phase-2 samples were heated to 450 °C using the same program. These samples are denoted as FCIM (450 °C), d_0.1_-FCIM (450 °C), d_0.15_-FCIM (450 °C), d_0.2_-FCIM (450 °C), Phase-1 (450 °C) and Phase-2 (450 °C).

PXRD patterns (2*θ* = 10° to 45°) were collected at room temperature using a Rigaku-RU 200B diffractometer (Rigaku, Tokyo, Japan) with Cu Kα radiation (λ = 1.54098 Å).

Solution ^1^H NMR spectra of all samples were recorded on a Bruker Avance III 500 MHz spectrometer (Bruker, Karlsruhe, Germany) at 293 K. Approximately 6 mg of the samples were dissolved in trifluoroacetic acid-d (CF_3_COOD; 750 μL). The residual carboxylic acid proton signal of CF_3_COOD was used as the internal reference for chemical shift calibration. All spectral data were processed using the MestreNova 14 Suite.

Fourier Transform Infrared (FT-IR) spectra were recorded on a Bruker INVENIO-S spectrometer (Bruker, Karlsruhe, Germany) using the KBr disk technique, and spectra were collected in transmission mode in the range of 4000–400 cm^−1^.

SEM images of samples were taken using a FEI QUANTA FEG 450 (FEI, Hillsboro, OR, USA). Prior to observations, all samples were coated with Pt for 120 s in a vacuum.

## 3. Results and Discussion

### 3.1. Morphology and Crystal Structure Evolution Induced by Ligand Substitution

**Figure 3:**
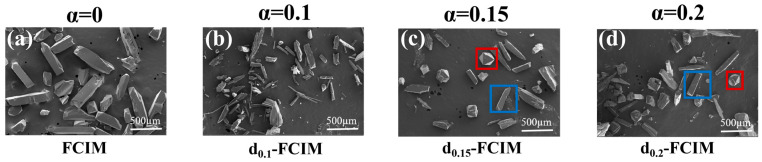
SEM micrographs of (**a**) FCIM (α = 0), (**b**) d_0.1_-FCIM (α = 0.1), (**c**) d_0.15_-FCIM (α = 0.15), and (**d**) d_0.2_-FCIM (α = 0.2). A coexistence of two distinct crystal morphologies is observed in (**c**,**d**). The red and blue squares represent crystals with two distinct morphologies: rod-like and rhombic octahedral structures.

As shown in the SEM images in Figure 3a, the FCIM sample without dmbIm added presents uniform rod-like particles with an average longitudinal dimension of approximately 500 µm. When the linker ratio *α* (= dmbIm/(dmbIm + Im)) is 0.1, the d_0.1_-FCIM sample (Figure 3b) displays a similar rod-like morphology with dimensions comparable to the pristine sample. In comparison, the d_0.15_-FCIM (*α* = 0.15) sample (Figure 3c) exhibits a coexistence of the original rod-like particles and newly emerged rhombic octahedra particles. This rhombic octahedra morphology is similar to that observed in the ZIF-4 with 1-methylimidazole doping [18]. While the size of the rod-like particles remains unchanged, the average dimension of the rhombic octahedra is observed to be approximately 300 µm. Upon further increasing the content of dmbIm, in the SEM images of d_0.2_-FCIM, the amount of the rhombic octahedra increases. This phenomenon indicates a progressive morphological transition driven by the increase in the substitution of Im with dmbIm in the synthesis.

**Figure 4:**
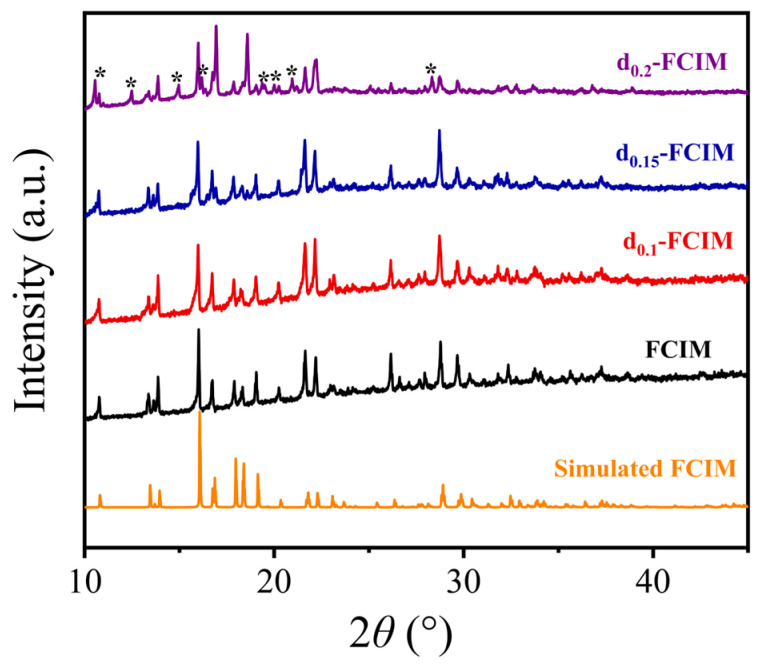
PXRD patterns of the FCIM (black), d_0.1_-FCIM (red), d_0.15_-FCIM (blue) and d_0.2_-FCIM (purple) with the simulated pattern of FCIM (orange). Asterisks (*) mark the peak differences observed between the simulated FCIM and the d_0.2_-FCIM.

The PXRD pattern of FCIM (Figure 4) exhibits characteristic Bragg peaks consistent with the pattern of simulated FCIM [6]. The diffraction peaks of the d_0.1_-FCIM and d_0.15_-FCIM samples are almost identical to those of the FCIM sample, indicating that the framework structure is well-maintained at low dmbIm concentration. Although distinct crystals with a new morphology in the d_0.15_-FCIM sample are evident in the SEM observations, their absence in the PXRD pattern suggests that they are present only in trace amounts, insufficient to generate obvious diffraction peaks [19]. However, as the α increases to 0.2 (d_0.20_-FCIM), some new diffraction peaks appeared, suggesting the formation of new phases in the d_0.2_-FCIM sample.

To investigate the chemical component of these FCIM samples, the ^1^H NMR measurements were performed (Figure S1). Apart from the signal at 11.50 ppm corresponding to the solvent CF_3_COOD [20], the spectrum of the FCIM sample exhibits two characteristic resonances at 8.39 ppm (signal 1) and 7.31 ppm (signal 2), both assigned to imidazole [6], confirming that FCIM contains the imidazole ligand exclusively. In contrast, for the series of samples synthesized by adding the secondary ligand of dmbIm (d_0.1_-FCIM, d_0.15_-FCIM, and d_0.2_-FCIM), there are three distinct signals observed at 8.57, 7.44, and 2.35 ppm (labeled as Signals 3–5). These resonances correspond to the characteristic of the dmbIm ligand [21], thereby providing spectroscopic evidence for the successful incorporation of dmbIm ligands into the framework. Furthermore, the intensity of the signal at 2.35 ppm exhibits a monotonic increase with dmbIm content. This trend confirms the gradual substitution of Im by dmbIm ligands in the Fe-ZIF.

In addition to the ^1^H NMR results, FT-IR measurement is employed to verify the presence of dmbIm specific functional groups [22,23,24]. As shown in Figure S2, compared to the FCIM sample, the spectra of d_0.1_-FCIM, d_0.15_-FCIM, and d_0.2_-FCIM display several new characteristic peaks. The peak at 990 cm^−1^ is attributed to the skeletal deformation of the benzene ring, serving as a diagnostic signature of the ortho-disubstituted benzene moiety within dmbIm. The signals at 1371 cm^−1^ are ascribed to the symmetric deformation vibration of the CH_3_ groups. Furthermore, the absorption bands at 2937 cm^−1^ and 2964 cm^−1^ correspond to the symmetric and asymmetric stretching vibrations of the CH_3_ groups, respectively. These new characteristic peaks further confirm the successful substitution of Im by dmbIm ligands in the Fe-ZIF crystalline lattice.

### 3.2. Effect of Ligand Substitution on Melting and Glass Formation in Fe-ZIF

**Figure 5:**
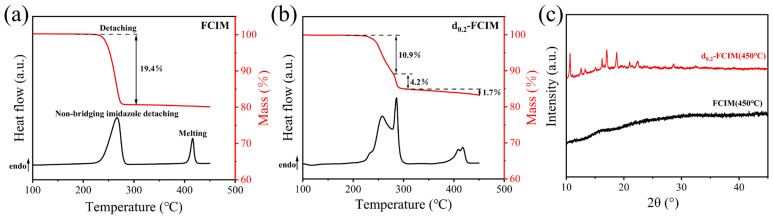
DSC and TGA curves of the FCIM (**a**) and the d_0.2_-FCIM (**b**) samples. As well as (**c**) the PXRD patterns of the FCIM (450 °C) and d_0.2_-FCIM (450 °C).

To investigate the impact of dmbIm substitution on the melting and glass formation of the Fe-ZIF samples, DSC and TGA were performed on the FCIM sample and the d_0.2_-FCIM sample (Figure 5). During DSC upsanning, the FCIM (Figure 5a) exhibits an endothermic peak in the temperature range from 218 °C to 289 °C, accompanied by a 19.4% mass loss, which is attributed to the detachment of non-bridging imidazole [25]. This process is due to the phase transition of the FCIM crystals from a structure constructed by mixed tetrahedral [Fe-Im_4_]/octahedral [Fe-Im_6_] units to a phase dominated exclusively by tetrahedral [Fe-Im_4_] units. The second endothermic peak from 395 °C to 428 °C without mass loss, which is attributed to the melting event [6,25]. Upon cooling, the melt-quenched FCIM (450 °C) formed its glassy state, evidenced by diffuse scattering in PXRD (Figure 5c) and a distinct glass transition (*T*_g_) at 192.5 °C during the second upscan (Figure S3).

In contrast, the d_0.2_-FCIM sample (Figure 5b) displays a more complex calorimetric behavior. At the temperature range from 200 °C to 314 °C, a non-symmetric endothermic peak is observed, corresponding to two-stage mass losses of 10.9% and 4.2%, respectively. At temperatures from 353 °C to 432 °C, two endothermic peaks occur without mass loss, which are the melting behavior. It indicates that there exist two meltable phases in the d_0.2_-FCIM sample. It could be the initial FCIM phase and the phase possessing the same FCIM crystalline structure but with dmbIm substitution. Notably, as shown in Figure 5c, the PXRD pattern of the d_0.2_-FCIM (450 °C) sample exhibits distinct diffraction peaks, implying there is also a non-meltable phase in the d_0.2_-FCIM sample. Considering the two crystalline particles with different morphologies observed in the d_0.2_-FCIM sample (Figure 3d), it is reasonable to infer that the two kinds of particles could be assigned to the meltable and non-meltable phases, respectively.

**Figure 6:**
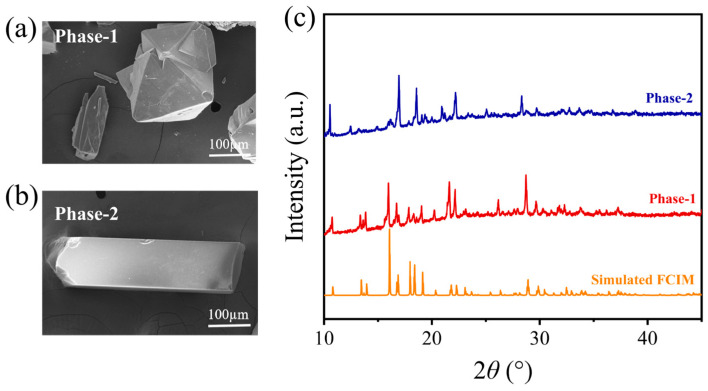
SEM micrographs of Phase-1 (**a**) and Phase-2 (**b**). (**c**) PXRD patterns of the Phase-1 (red) and Phase-2 (blue) with the simulated pattern of FCIM (orange).

In order to investigate the structural difference between the meltable and non-meltable phases. The crystals in the d_0.2_-FCIM sample are physically separated based on their distinct morphologies. The rod-like crystals were designated as Phase-1 (Figure 6a), while the rhombic octahedral crystals were designated as Phase-2 (Figure 6b). The PXRD patterns (Figure 6c) reveal that Phase-1 retains the crystalline structure of the FCIM. In contrast, the diffraction pattern of Phase-2 corresponds perfectly to the previously unidentified peaks observed in the d_0.2_-FCIM sample. This well suggests that the two particles with different morphologies in the d_0.2_-FCIM sample are two phases with distinct structures.

**Figure 7:**
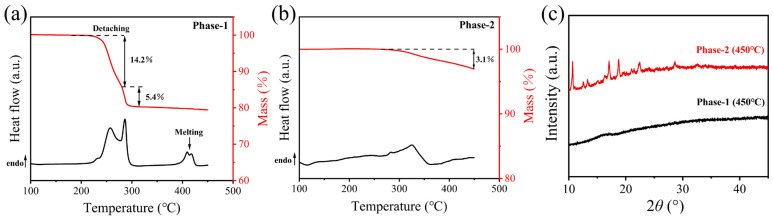
DSC and TGA curves of the phase-1 (**a**) and the phase-2 (**b**). (**c**) PXRD patterns of the corresponding heat-treated samples of Phase-1 (450 °C) and Phase-2 (450 °C) obtained after the first DSC upscanning.

Subsequently, the DSC measurements for the Phase-1 and Phase-2 were performed. As shown in Figure 7a, Phase-1 exhibits a non-symmetric endothermic peak at the temperature range of 200 °C to 314 °C, accompanied by mass losses of 14.2% and 5.4%, respectively. At 353 °C to 432 °C, two endothermic peaks appear, indicating melting. This DSC upscanning curve is almost identical to that of the d_0.2_-FCIM sample (Figure 5b). Moreover, upon melt-quenching, Phase-1 shows a clear glass transition (*T*_g_ = 192.1 °C) (Figure S4) and no obvious diffraction peaks in PXRD (Figure 7c), confirming the formation of its glass. This proves that Phase-1 is the meltable phase in the d_0.2_-FCIM sample. Conversely, during DSC upscanning to 450 °C, Phase-2 (Figure 7b) exhibits no clear melting peak, only a minor mass loss (3.1%), indicating excellent thermal stability. In addition, the PXRD pattern of Phase-2 remains crystalline even after heating to 450 °C (Figure 7c). These diffraction peaks perfectly match the residual signals of the d_0.2_-FCIM (450 °C) (Figure 5c), implying that Phase-2 is the non-meltable phase in the d_0.2_-FCIM sample.

### 3.3. Characterizations of the Meltable and Non-Meltable Phases

**Figure 8:**
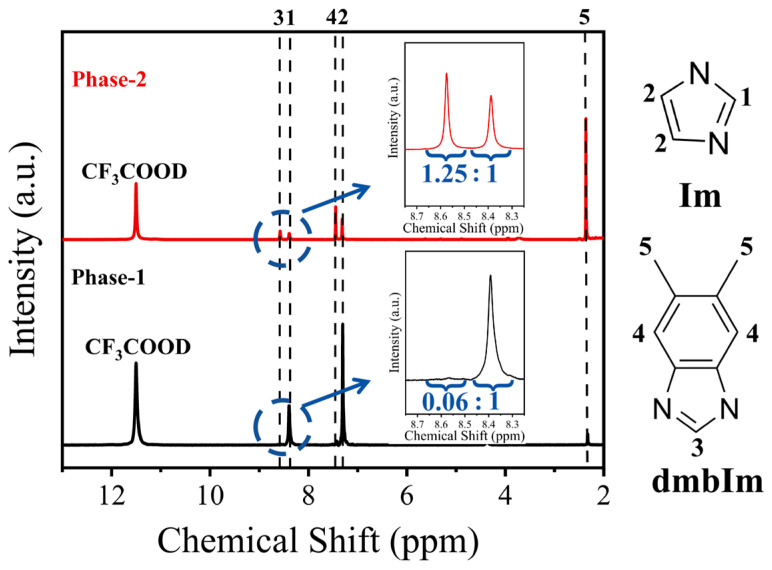
Solution ^1^H NMR spectra of the Phase-1 and the Phase-2. The inset is a magnification for the integration of the characteristic peaks assigned to the Im and dmbIm ligands.

To detect the specific chemical compositions of the Phase-1 and Phase-2 samples, ^1^H NMR spectroscopy was conducted (Figure 8). Apart from the residual solvent resonance of CF_3_COOD at 11.50 ppm, the spectra of both phases exhibit the same set of five characteristic signals. Similar to the ^1^H NMR results shown in Figure S1, the peaks at 8.39 ppm (Signal 1) and 7.31 ppm (Signal 2) are ascribed to the imidazole ligand, while the resonances at 8.57 ppm (Signal 3), 7.44 ppm (Signal 4), and 2.35 ppm (Signal 5) correspond to dmbIm. The molar ratio of the ligands in each phase can be determined by integrating the characteristic peaks [26]. Since Signal 1 (Im) and Signal 3 (dmbIm) each represent a single proton site, the ratio of their integrated areas directly yields the molar ratio of Im to dmbIm [21]. The result shows a distinct compositional difference: Phase-1 possesses a low Im:dmbIm ratio of 1:0.06, whereas Phase-2 has a ratio of 1:1.25.

**Figure 9:**
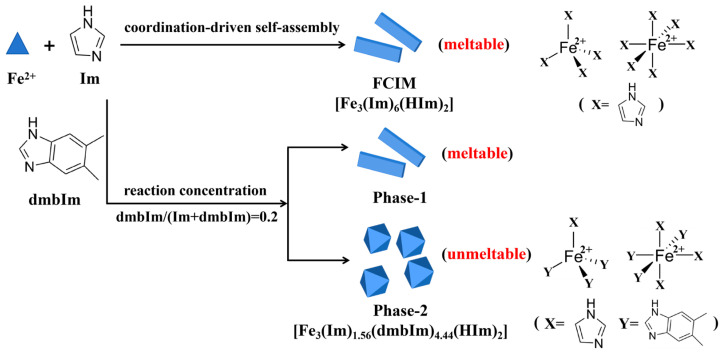
Schematic diagram of the effect of dmbIm substitution on the crystal structure and morphology of Fe-ZIF.

The ^1^H NMR measurement (Figure 8) suggests that the incorporation threshold of dmbIm in the Fe_3_(Im)_6_(HIm)_2_ structure is limited. Based on a series of characterizations conducted in this work, the overall impact of dmbIm substitution on the crystal structure and morphology of Fe-ZIF was analyzed (Figure 9). In such a substitution level, the Phase-1 retains the crystalline lattice and morphology of FCIM crystals [26], and also maintains the ability to melt. As reported previously [27], the structure of FCIM is constructed by tetrahedral [FeIm_4_] and octahedral [FeIm_6_] units linked via imidazolate bridges. The octahedral [FeIm_6_] units possess two terminal imidazole molecules in trans-configuration. During heating, the removal of terminal imidazole molecules at 200 °C~314 °C (Figure 5a) triggers a conversion of the Fe^2+^ coordination environment from octahedral to tetrahedral centers, i.e., a structural transition from Fe_3_(Im)_6_(HIm)_2_ to Fe(Im)_2_. As shown in Figure 3a and Figure 5a, both FCIM and Phase-1 samples exhibit a mass loss of ~19% during terminal imidazole release. So, it is highly probable that dmbIm substitutes the non-terminal imidazole sites, and hence the dmbIm ligands are retained in the crystal structure after the structural transformation [28]. Moreover, it is clearly observed that Phase-1 possesses two stages of mass loss and a bimodal-resolvable endothermic peak (Figure 7a), indicating that the incorporation of a large-sized ligand of dmbIm affects the release process of terminal imidazole and the melting point. This could be ascribed to the steric hindrance effect induced by a large-size ligand, consistent with previous publications [29,30].

In contrast, based on the Im:dmbIm molar ratio of 1:1.25, the molecular formula of Phase-2 can be represented as Fe_3_(Im)_1.56_(dmbIm)_4.44_(HIm)_2_. Thus, Phase-2 represents a phase with a high ratio of dmbIm substitution, which makes it become a new phase much differing from FCIM crystals. And this Phase-2 is non-meltable. Considering the coordination structure of the Fe^2+^ center, we infer that, in Phase-2, the dmbIm ligands substitute the bridged Im ligand (i.e., the non-terminal Im linkers), yielding a theoretical stoichiometry of Fe_3_(Im)_1.5_(dmbIm)_4.5_(HIm)_2_, which is in excellent agreement with the measured result of Fe_3_(Im)_1.56_(dmbIm)_4.44_(HIm)_2_ derived from ^1^H NMR results.

### 3.4. Effect Mechanism of Ligand Substitution on the Meltability of Fe-ZIF

In this work, secondary ligands of dmbIm were introduced in the FCIM structure to substitute Im ligands. When the substitution ratio is low (α = 0.1 and 0.15), the incorporation of dmbIm into the FCIM crystal structure does not induce structural reconstruction. The dmbIm ligands have a similar local coordination environment as the Im ligands [31]. In this way, the methyl groups in dmbIm act as electron-donating moieties, boosting the electron density at the coordination units and favoring the formation of Fe-dmbIm bonds [21]. That is the formation of Phase-1. This is verified by the retention of the parent crystal structure in the d_0.1_-FCIM and d_0.15_-FCIM samples. When the substitution ratio reaches 0.2, the accumulated strain from the bulky methyl groups destabilizes the parent lattice, inducing a phase transition to Phase-2 [18].

In Phase 1, the partial substitution of Im by dmbIm creates a distortion of the tetrahedral [Fe-ligands], inducing a decrease in symmetry of structural units. According to the theory of melting, such crystals with lower symmetry are predisposed to melting because they possess higher configurational degrees of freedom [32]. Specifically, the introduction of dmbIm disrupts the uniform cohesive network of the parent FCIM. This distortion in structural units makes the coordination bonds dissociate at a lower temperature [29]. However, the effect of steric hindrance induced by the incorporation of large-sized ligands causes the melting temperature to increase. Therefore, the competition between the distortion of structural units and steric hindrance contributes to the occurrence of secondary melting in the DSC curve of Phase-1.

Conversely, the absence of melting in Phase-2 can be rationalized by the enhancement of cohesive interactions in the framework. The high concentration of dmbIm leads to a robust framework [10]. The saturation of dmbIm ligands in Phase-2 creates a “locked” lattice where steric hindrance effects reinforce the structural rigidity rather than promoting fluidity [5]. Consequently, the lattice lacks the necessary microscopic fluctuations required to trigger the melting. The energy required to dissociate these reinforced coordination bonds exceeds the thermal stability of the organic linkers [33], meaning the framework decomposes before it can access a liquid state [34]. Thus, Phase-2 behaves as a non-meltable solid.

## 4. Conclusions

In summary, we synthesized a series of Fe-ZIF by substituting the Im ligands with secondary dmbIm ligands via a solvent-free strategy. With the increase in the substitution ratio, the new phase forms, evidenced by the newly emerged XRD diffraction peaks and crystal particles with new morphology. Specifically, at the dmbIm/(Im + dmbIm) ratio of 0.2 in synthesis, the product possesses the coexistence of a meltable Phase-1 and a non-meltable Phase-2. The differences in chemical composition and structure between the Phase-1 and Phase-2 were systematically investigated. The partial substitution of Im by dmbIm in Phase-1 maintains the structure lattice, which is meltable, but affects the melting behavior due to the distortion of structural units and steric hindrance effects. Conversely, the steric locking and high structural rigidity in Phase-2 preclude melting by shifting the theoretical melting point beyond the decomposition threshold. These findings offer an insight into how ligand substitution alters the melting of ZIFs, suggesting a practical pathway for the synthesis of functional ZIF glasses.

## Data Availability

The original contributions presented in this study are included in the article/Supplementary Materials. Further inquiries can be directed to the corresponding authors.

## Supplementary Materials

The following supporting information can be downloaded at: https://www.mdpi.com/article/10.3390/ma19101926/s1, Figure S1: Solution ^1^H NMR spectra of the FCIM (black), d_0.1_-FCIM (red), d_0.15_-FCIM (blue) and d_0.2_-FCIM (purple); Figure S2: FT-IR transmission spectra of the FCIM (black), d_0.1_-FCIM (red), d_0.15_-FCIM (blue) and d_0.2_-FCIM (purple) in the range of: (a) full-range spectrum (b) 3000–2900 cm^−1^, (c) 1410–1340 cm^−1^, (d) 1010–950 cm^−1^; Figure S3: DSC and TGA curves of the FCIM glass; Figure S4: DSC and TGA curves of the Phase-1 glass.
