# Supplementary material for: Effect of Ligand Substitution on the Formation of the Meltable Fe-ZIF
Source: Materials (Basel). 2026 May 8;19(10):1926. doi: 10.3390/ma19101926 (PMC13208247; doi:10.3390/ma19101926)
Supplement: Supplementary file 1 [file materials-19-01926-s001.zip › materials-4275307-supplementary.pdf]

materials

Supplementary Materials

Effect of ligand substitution on the formation of the meltable Fe-ZIF

Liuyang Zheng [a,†], Chaohui Guo [a,†], Zijuan Du [a], Juan Han [b], Ang Qiao [a,*], De Fang [b,*], Haizheng Tao [a]

[a] State Key Laboratory of Silicate Materials for Architectures, Wuhan University of Technology, Wuhan 430070, China
[b] School of Materials Science and Engineering, Wuhan University of Technology, Wuhan, 430070, China
† These authors contributed equally to this work.
* Corresponding authors: qiaoang@whut.edu.cn; fangde@whut.edu.cn

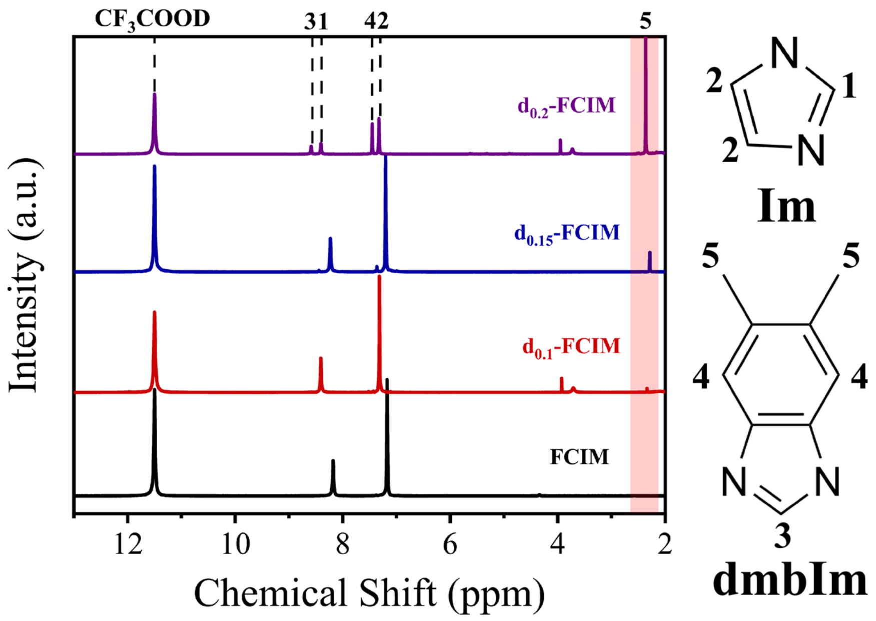

Figure S1. Solution ^{1}H NMR spectra of the FCIM (black), $d_{0.1}$-FCIM (red), $d_{0.15}$-FCIM (blue) and $d_{0.2}$-FCIM (purple).

 https://doi.org/10.3390/ma19101926

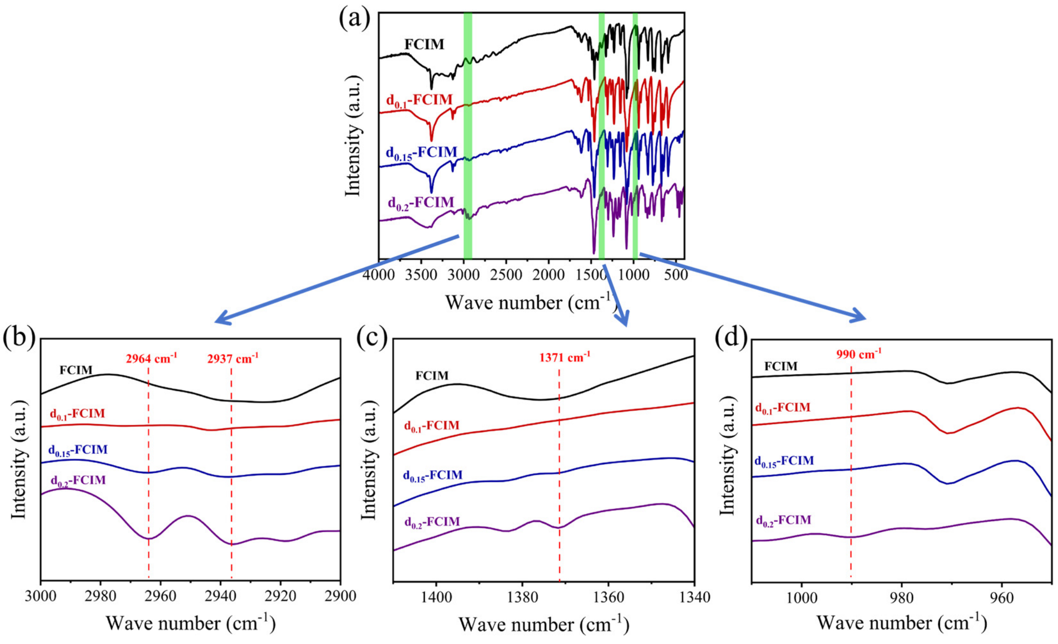

Figure S2. FT-IR transmission spectra of FCIM (black), $d_{0.1}$-FCIM (red), $d_{0.15}$-FCIM (blue) and $d_{0.2}$-FCIM (purple) in the range of: (a) full-range spectrum, (b) 3000-2900 cm^{-1}, (c) 1410-1340 cm^{-1}, and (d) 1010-950 cm^{-1}.

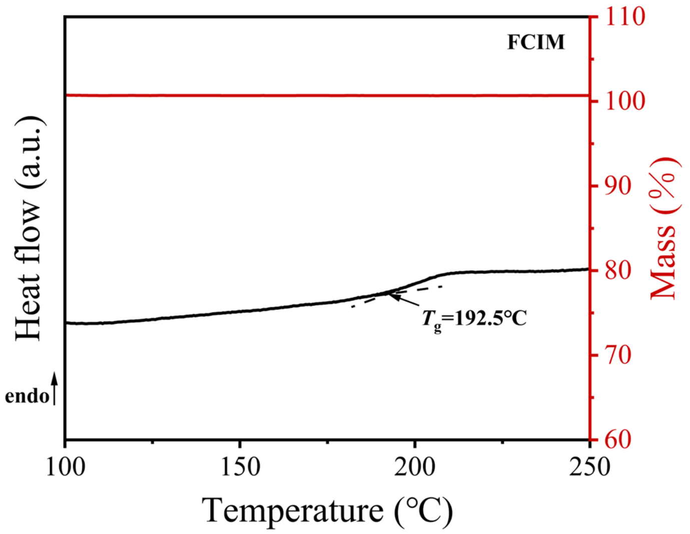

Figure S3. DSC and TGA curves of the FCIM glass.

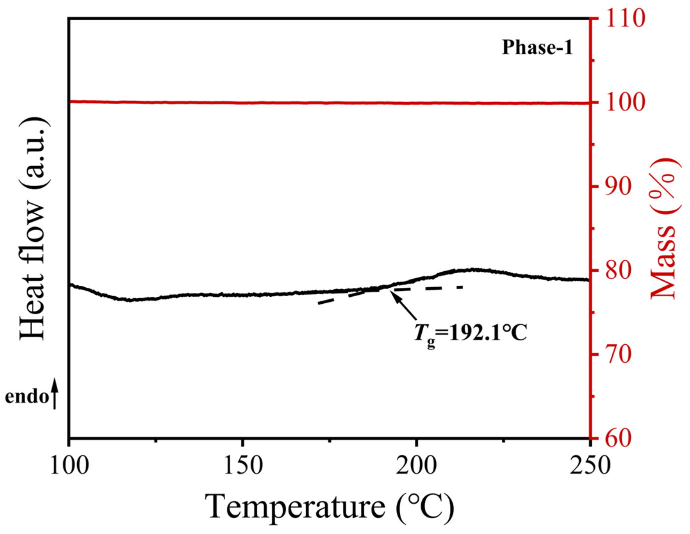

Figure S4. DSC and TGA curves of the Phase-1 glass.
